# Achilles’ Heel of currently approved immune checkpoint inhibitors: immune related adverse events

**DOI:** 10.3389/fimmu.2024.1292122

**Published:** 2024-02-12

**Authors:** Ting Yan, Lun Yu, Jiwen Zhang, Yun Chen, Yilan Fu, Jingyi Tang, Dehua Liao

**Affiliations:** ^1^ Department of Pharmacy, Hunan Cancer Hospital, The Affiliated Cancer Hospital of Xiangya School of Medicine, Central South University, Changsha, China; ^2^ Department of Positron Emission Tomography–Computed Tomography (PET-CT) Center, Chenzhou No. 1 People’s Hospital, Chenzhou, China; ^3^ School of Pharmacy, University of South China, Hengyang, China

**Keywords:** PD-1 inhibitors, PD-L1 inhibitors, CTLA-4 inhibitors, immune-related adverse events, ICIs, ICIS

## Abstract

Immunotherapy has revolutionized the cancer treatment landscape by opening up novel avenues for intervention. As the use of immune checkpoint inhibitors (ICIs) has exponentially increased, so have immune-related adverse events (irAEs). The mechanism of irAEs may involve the direct damage caused by monoclonal antibodies and a sequence of immune responses triggered by T cell activation. Common side effects include dermatologic toxicity, endocrine toxicity, gastrointestinal toxicity, and hepatic toxicity. While relatively rare, neurotoxicity, cardiotoxicity, and pulmonary toxicity can be fatal. These toxicities pose a clinical dilemma regarding treatment discontinuation since they can result in severe complications and necessitate frequent hospitalization. Vigilant monitoring of irAEs is vital in clinical practice, and the principal therapeutic strategy entails the administration of oral or intravenous glucocorticoids (GSCs). It may be necessary to temporarily or permanently discontinue the use of ICIs in severe cases. Given that irAEs can impact multiple organs and require diverse treatment approaches, the involvement of a multidisciplinary team of experts is imperative. This review aims to comprehensively examine the pathogenesis, clinical manifestations, incidence, and treatment options for various irAEs.

## 1 Introduction

Immunotherapy has emerged as a promising avenue for new cancer treatments by boosting the patient’s immune system (1). Immune checkpoint inhibitors (ICIs) such as those targeting programmed cell death protein 1 (PD-1) or its primary ligand (PD-L1), as well as the cytotoxic T lymphocyte-associated antigen 4 (CTLA-4) signaling, have demonstrated encouraging therapeutic effects against various types of solid tumors.

Ipilimumab was the first CTLA-4 inhibitor approved by the US Food and Drug Administration (2). It was followed by PD-1 inhibitors (e.g., pembrolizumab, nivolumab, and cemiplimab) and PD-L1 inhibitors (e.g., atezolizumab, durvalumab, and avelumab) have also been approved for a variety of indications. The National Medical Products Administration has approved an expanded range of drugs in this category. Currently, there are 10 PD-1 inhibitors (e.g., pembrolizumab, nivolumab, toripalimab, sintilimab, camrelizumab, tislelizumab, penpulimab, zimberelimab, serplulimab, and adebrelimab). Additionally, there are 4 PD-L1 inhibitors (e.g., atezolizumab, durvalumab, envafolimab, and sugemalimab). Furthermore, there were CTLA-4 inhibitors (ipilimumab and tremelimumab) and a combination inhibitor of PD-1 and CTLA-4 (cadonilimab).

Whether solid or non-solid tumors, ICIs play a vital role in cancer treatment, due to their well-established clinical benefits. The utilization of these agents is expected to increase significantly in the upcoming years (3). ICIs work by interacting with immune cells through signaling pathways, impairing their ability to recognize and eliminate cancer cells (4). Although effective against cancer, this approach can also result in immune-related adverse events (irAEs), mainly affecting the skin, endocrine glands, liver, lungs, gut, and potentially other organs. This susceptibility represents a significant drawback of this particular therapeutic agent, known as the Achilles’ Heel of immunotherapy (5). Understanding the underlying mechanisms is essential for prompt diagnosis and, more importantly, appropriate therapeutic management. Therefore, this review aims to present the pathogenesis, clinical manifestations, incidence, and treatment strategies of various irAEs through 49 clinical trials from ICIs encompassing solid and non-solid tumors, retrospective analyses, and case reports. Hopefully, this will help provide a deeper understanding of irAEs.

## 2 Mechanism

The emergence and intensity of irAEs could potentially be influenced by various immune mechanisms. Existing evidence suggests that during the later stages of the immune response (6), ICIs can facilitate the infiltration of T-cells into peripheral tissues, which in turn, might explain the occurrence of irAEs in PD-1/PD-L1 blockade (7). Furthermore, ICIs have been shown to reduce the survival and inhibitory function of regulatory T (Treg) cells while concurrently augmenting cytokine production (8).

**Figure 1:**
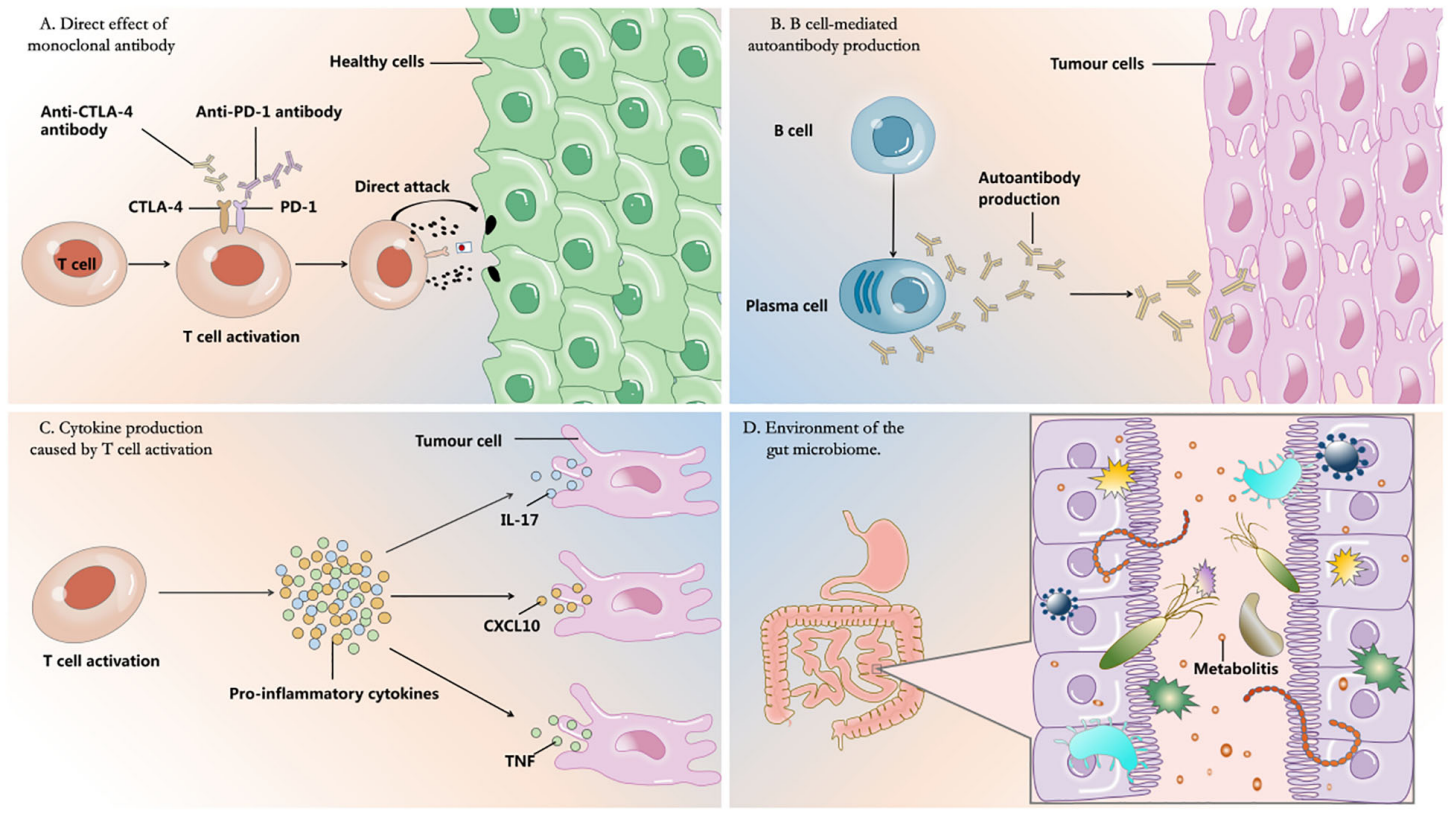
Four proposed mechanisms for the development of irAEs.

Several proposed mechanisms have been put forth to elucidate irAEs (**Figure 1**).

One such mechanism revolves around the direct effect of monoclonal antibodies. It has been postulated that some irAEs may arise due to the complement-mediated direct injury caused by monoclonal antibody therapies (9). PD-L1, a molecule mainly expressed in the endothelium of the myocardium, plays a pivotal role in regulating immune-mediated cardiac injuries (10). In a patient who succumbed to myocarditis following combination therapy of ICIs, there were observations of a tenfold increase in PD-L1 expression in the cardiac tissue as compared to unaffected muscle tissue (11).

Another crucial aspect involving irAEs is the heightened production of autoantibodies by B cells following immunotherapy. It is possible that individuals who develop grade ≥3 irAEs, might have an increased presence of self-reactive B cells in the bloodstream after undergoing immunotherapy (12). Through immunotherapy-induced activation, T cells foster greater interactions with B cells, subsequently leading to the production of autoantibodies. For instance, the interactions between follicular T cells and B cells in germinal centers play a vital role in the development of humoral immunity, and any disruptions in these interactions have been linked to autoimmune diseases (13). Research has demonstrated that patients with antithyroid antibodies experience more severe thyroid dysfunction when subjected to PD-1 therapy (14).

Thirdly, the occurrence of irAEs can be elucidated by the fact that the activation of T cells stimulates the production of cytokines. Research has demonstrated that the depletion of Treg cells, which play a crucial role in maintaining peripheral tolerance, is observed during the administration of ICIs and contributes to the manifestation of irAEs (15). This depletion is hypothesized to transpire through the differentiation process of T helper 17 (Th17) cells into Treg cells (16–18), subsequently leading to an imbalance between Treg cells and Th17 cells which has been implicated in the development of irAEs (19). Th17 cells are renowned for their secretion of pro-inflammatory cytokines such as IL-17A, IL-21, and IL-22, which have been implicated in the pathogenesis of autoimmune diseases like rheumatoid arthritis and psoriatic arthritis (20). However, the influence of other pro-inflammatory cytokines on the manifestation of irAEs has not been comprehensively explored. Nonetheless, the analysis of serum cytokine levels has demonstrated a significant elevation in various levels of several pro-inflammatory cytokines among irAE patients, including IL-1Ra, CXCL10, and TNF-α, as well as soluble IL-2 receptors (21). A documented case report has proposed that the use of anti-TNF agents effectively manages irAEs in patients undergoing ICI therapy, suggesting a potential role of TNF in the development of irAEs (22).

Finally, recent research has revealed that the gut microbiota, specifically *Bifidobacterium*, *Bacteroides fragilis*, and *Akkermansia muciniphia*, play a vital role in enhancing the effectiveness of ICIs and impacting their toxicity (23–25). This is accomplished by modifying metabolites derived from nutrients in the host, maintaining the integrity of the gut mucosa barrier, and participating in immune-modulation (26). Various techniques for manipulating A. muciniphila in the gut microbiota have been described, such as fecal microbiota transplantation (FMT), probiotics, prebiotics, and dietary interventions( (27). For example, in a study conducted by Wang Y. et al (28), successful treatment of immune-related colitis was achieved by utilizing FMT to restore the gut microbiota of oncology patients, suggesting that reshaping the gut microbiota could alleviate immune-related colitis. Additionally, promising results have emerged from recent clinical trials highlighting the significance of *Akkermansia* in immunotherapy for non-small cell lung cancer (NSCLC) (29). Notably, individuals with higher levels of *Bacteroides fragilis* are found to have a reduced risk of colitis, while those with an abundance of *Firmicutes* face an increased risk (30, 31).

Here, in **Figure 1**, we depict the immune mechanisms driving irAEs including: A. direct effect of monoclonal antibody; B. B cell-mediated autoantibody production; C. cytokine production caused by T cell activation; D. environment of the gut microbiome.

## 3 Immune-related adverse events

ICIs primarily target the immune system for combatting cancer. However, this mechanism unfortunately results in autoimmune-like toxicities, that are exclusive to ICIs and not observed with other targeted agents or cytotoxic chemotherapy (32). These toxicities have the potential to affect various tissues or organs such as the skin, endocrine system, liver, gastrointestinal tract, lungs, and rheumatoid/skeletal muscle. Although less common, the nervous system, blood, kidneys, heart, and eyes may also be affected. In rare instances, transfusion reactions may occur. While the majority of irAEs are mild and reversible, they can arise at any point, except for long-term endocrine irAEs (33–35). Severe irAEs are infrequent but can have significant consequences, especially when they impact the pericardium, lungs, and nervous system (36–38). A systematic review of 50 trials encompassing 5071 patients discovered that the median rate of grade 3/4 irAEs was 21% (39). The occurrence of irAEs caused by ICIs in 49 clinical trials involving solid or non-solid tumors is depicted in **Table 1**.

**Table 1:** The incidence of immune-related adverse events in clinical trials with immune checkpoint inhibitors.

Treatment(patients)	Clincal trials	Phase	Tumor Types	IrAEs (any grade)	IrAEs (grade 3-5)	Reference
Nivolumab(n=337)	CheckMate 078	III	NSCLC	**Total:64%** Rash(12%), pruritus(8%), ALT elevation(9%), AST elevation(9%), thyroid disorder(9%), hypothyroidism(4%), GGT elevation(4%)	**Total:10%** Rash(1%), pneumonitis(1%), interstitial lungdisease(1%), ALT elevation(<1%), AST elevation(<1%), GGT elevation(<1%)	(40)
Nivolumab(n=418)	CheckMate 017&057	III	NSCLC	**Total:68%** Fatigue(17%), nausea(11%), decreased appetite(11%), asthenia(11%), diarrhea(9%), rash(8%), pruritus(7%), hypothyroidism(6%), arthralgia(6%)	**Total:11%** Fatigue(1%), pneumonitis(1%), diarrhea(1%), rash(<1%), nausea(<1%)	(41)
Nivolumab plus ipilimumab(n=300)	CheckMate 743	III	Malignant pleural mesothelioma	**Total:80%** Diarrhea(21%), pruritus(16%), rash(14%), fatigue(14%), hypothyroidism(11%), nausea(10%), decreased appetite(10%)	**Total:30%** Increased lipase(4%), increased lipase(4%), diarrhea(3%), increased amylase(2%), decreased appetite(1%), fatigue(1%), neutropenia(1%), thrombocytopenia(1%)	(42)
Nivolumab plus ipilimumab(n=404)	CheckMate 914	III	Renal cell carcinoma	**Total:59%** Pruritus(31%), fatigue(30%), diarrhea(24%), rash(21%), headache(17%), nausea(17%), hyperthyroidism(16%), arthralgia(16%), hypothyroidism(16%), decreased appetite(13%), cough(12%), asthenia(11%)	Diarrhea(4%), fatigue(1%), rash(1%)	(43)
Nivolumab(n=330)	ATTRACTION-2	III	Gastric or gastro-esophageal junction cancer	**Total:43%** Pruritus(9%), diarrhea(7%), rash(6%), fatigue(5%), decreased appetite(5%), nausea(4%), malaise(4%)	**Total:10%** Diarrhea(1%), fatigue(1%), decreased appetite(1%), AST increased(1%)	(44)
Nivolumab plus capecitabine/leucovorin/fluorouracil and oxaliplatin(n=782)	CheckMate 649	III	Gastric, gastro esophageal junction, and esophageal adenocarcinoma	**Total:94%** Nausea(41%), diarrhea(32%), peripheral neuropathy(28%), fatigue(26%), anaemia(26%), vomiting(25%), neutropenia(24%), decreased appetite(20%), thrombocytopenia(20%), PLT decreased(20%)	**Total:60%** Neutropenia(15%), NEU decreased(11%), anaemia(6%), lipase increased(6%), diarrhea(4%), peripheral neuropathy(4%), peripheral neuropathy(4%), fatigue(4%),nausea(3%)	(45)
Pembrolizumab(n=154)	KEYNOTE-024	III	NSCLC	**Total:77%** Diarrhea(16%), fatigue(14%), pyrexia(12%), pruritus(12%), rash(10%), nausea(10%), anorexia/decreased appetite(10%)	**Total:31%** Diarrhea(4%), fatigue(2%), rash(1%), anaemia(1%)	(46)
Pembrolizumab(n=637)	KEYNOTE-042	III	NSCLC	**Total:63%** Hypothyroidism(11%), fatigue(8%), pruritus(7%), rash(7%), ALT increased(7%), pneumonitis(7%), AST increased(6%), decreased appetite(6%), hyperthyroidism(6%), anaemia(6%)	**Total:18%** Pneumonitis(3%), ALT increased(1%), AST increased(1%), decreased appetite(1%), anaemia(1%), diarrhea(1%)	(47)
Pembrolizumab(n=314)	KEYNOTE-181	III	Esophageal cancer	**Total:64%** Fatigue(12%), hypothyroidism(11%), decreased appetite(9%), nausea(7%), asthenia(7%), diarrhea(5%)	**Total:18%** Decreased appetite(1%), diarrhea(1%), asthenia(1%), fatigue(1%), anemia(1%)	(48)
Pembrolizumab(n=110)	KEYNOTE-427	II	Renal cell carcinoma	**Total:80%** Pruritus(27%), fatigue(25%), diarrhea(19%), rash(15%), arthralgia(13%), hypothyroidism(10%)	Diarrhea(4%), AST increased(2%), asthenia(2%)	(49)
Pembrolizumab(n=300)	KEYNOTE-048	III	Head and neck squamous cell carcinoma	Fatigue(28%), anaemia(21%), constipation(20%), hypothyroidism(18%), nausea(16%), diarrhea(15%), weight decreased(15%), decreased appetite(15%)	Anaemia(5%), fatigue(3%), weight decreased(2%), hypokalaemia(2%), diarrhea(1%), asthenia(1%), mucosal inflammation(1%), decreased appetite(1%), rash(1%)	(50)
Pembrolizumab plus platinum and 5-fluorouracil(n=276)	KEYNOTE-048	III	Head and neck squamous cell carcinoma	Anaemia(58%), nausea(51%), constipation(37%), neutropenia(34%), fatigue(34%), vomiting(33%), mucosal inflammation(31%), decreased appetite(29%), Thrombocytopenia(29%), diarrhea(28%)	Anaemia(25%), neutropenia(18%), NEU decreased(11%), mucosal inflammation(10%), thrombocytopenia(9%), Stomatitis(8%), fatigue(7%), hypokalaemia(7%), nausea(6%)	(50)
Cemiplimab(n=193)	EMPOWER-CSCC-1	II	Cutaneous squamous cell carcinoma	**Total:99%** Fatigue(35%), diarrhea(27%), nausea(24%), pruritus(21%), arthralgia(18%), cough(17%), rash(17%), constipation(15%), vomiting(13%)	**Total:49%** Anemia(4%), fatigue(3%), diarrhea(1%), constipation(1%), vomiting(1%), arthralgia(1%), rash(1%), rash maculo-papular(1%)	(51)
Cemiplimab(n=355)	EMPOWER-Lung	III	NSCLC	**Total:57%** ALT increased (6%), AST increased (6%), decreased appetite(5%), anaemia(5%), rash(5%), diarrhea(4%), nausea(4%), arthralgia(4%), fatigue(4%)	**Total:14%** ALT increased (1%), AST increased(1%), anaemia(1%), rash(1%), fatigue(1%), increased blood alkaline phosphatase(1%), increased weight(1%), dyspnea(1%), neutropenia(1%)	(52)
Toripalimab plus gemcitabine-cisplatin(n=146)	JUPITER-02	III	Nasopharyngeal carcinoma	**Total:100%** Leukopenia(91%), anemia(88%), neutropenia(86%), nausea(69%), vomiting(67%), thrombocytopenia(64%), decreased appetite(53%), constipation(39%), AST increased(38%), ALT increased(36%)	**Total:89%** Leukopenia(62%), neutropenia(58%), anemia(47%), thrombocytopenia(33%), pneumonia(10%), natremia(9%), hyponatremia(9%), lymphopenia(9%), hypokalemia(7%)	(53)
Toripalimab plus paclitaxel and cisplatin(n=257)	JUPITER-06	III	Esophageal squamous cell carcinoma	**Total:99%** Anemia(78%), leukopenia(68%), neutropenia(67%), nausea(43%), neuropathy peripheral(40%), vomiting(40%), decreased appetite(39%), alopecia(35%), weight decreased(29%), hypoproteinemia(25%)	**Total:73%** Neutropenia(42%), leukopenia(20%), anemia(11%), pneumonia(6%), fatigue(4%), hyponatremia(4%), weight decreased(3%), rash(3%), hypokalemia(3%)	(54)
Sintilimab plus pemetrexed and platinum(n=266)	ORIENT-11	III	NSCLC	**Total:43%** Hypothyroidism(7%), rash(6%), AST increased(6%), ALT increased(6%), increased thyroid stimulating hormone(5%), hyperthyroidsm(5%), diarrhea(5%), pneumonitis(3%), decreased thyroid stimulating hormone(3%), increased amylase(3%)	**Total:6%** Pneumonitis(1%), increased amylase(1%)	(55)
Sintilimab plus platinum and gemcitabine(n=179)	ORIENT-12	III	NSCLC	Anemia(93%), WBC decreased(89%), NEU decreased(83%), platelet count decreased(73%), nausea(40%), asthenia(34%), vomiting(32%), decreased appetite(32%), constipation(31%)	NEU decreased(49%), PLT decreased(45%), WBC decreased(36%), anemia(34%), infectious pneumonitis(14%), hyponatremia(6%), asthenia(2%), vomiting(2%), rash(2%), hemoptysis(2%)	(56)
Sintilimab plus cisplatin and paclitaxel(n=327)	ORIENT-15	III	Esophageal squamous cell carcinoma	**Total:98%** Anemia(75%), WBC decreased(64%), NEU decreased(62%), nausea(47%), vomiting(34%), asthenia(33%), decreased appetite(28%), hypoaesthesia(23%), PLT decreased(21%)	**Total:60%** Asthenia(45%), NEU decreased(30%), WBC decreased(17%), anemia(13%, decrease in lymphocyte count(5%), PLT decreased(3%), decrease in lymphocyte count(3%), blood pressure increased(3%), pneumonia(3%)	(57)
Sintilimab plus bevacizumab biosimilar(n=380)	ORIENT-32	III	Hepatocellular carcinoma	**Total:99%** Proteinuria(42%), PLT decreased(41%), increased AST(36%), hypertension(32%), increased blood bilirubin(29%), increased ALT(26%), WBC decreased(20%), pyrexia(17%), hypoalbuminaemia(17%), asthenia(16%)	**Total:55%** Hypertension(14%), PLT decreased(8%), proteinuria(5%), increased blood bilirubin(5%), increased γ-glutamyltransferase(5%), elevated blood pressure(3%), abnormal liver function(3%), increased conjugated bilirubin(3%), NEU decreased(2%)	(58)
Camrelizumab plus carboplatin and pemetrexed(n=205)	CameL	III	NSCLC	RECCP(78%), NEU decreased(71%), WBC decreased(71%), anaemia(66%), anaemia(66%), PLT decreased(46%), AST increased(45%), ALT increased(43%), nausea(36%), asthenia(31%), decreased appetite(30%)	NEU decreased(38%), WBC decreased(20%), anaemia(19%), PLT decreased(17%), ALT increased(5%), lymphocyte count decreased(4%), bone marrow toxicity(4%), asthenia(3%), GGT increased(3%)	(59)
Camrelizumab plus carboplatin and paclitaxel(n=193)	CameL-sq	III	Squamous NSCLC	WBC decreased(79%), NEU decreased(78%), RCCEP(69%), anemia(63%), PLT decreased(41%), asthenia(33%), hypoaesthesia(30%), decreased appetite(28%), nausea(24%)	NEU decreased(55%), WBC decreased(30%), anemia(10%), PLT decreased(7%), lymphocyte count decreased(4%), pneumonia(4%), RCCEP(2%), asthenia(2%), ALT increased(2%)	(60)
Camrelizumab plus gemcitabine and cisplatin(n=134)	CAPTAIN-1st	III	Nasopharyngeal carcinoma	WBC decreased(96%), anaemia(95%), NEU decreased(95%), PLT decreased(80%), nausea(70%), decreased appetite(64%), RECCP(58%), vomiting(56%), asthenia(49%), hypothyroidism(46%), constipation(45%)	WBC decreased(66%), NEU decreased(64%), PLT decreased(40%), anaemia(39%), lymphocyte count decreased(19%), hyponatraemia(10%), hypokalaemia(7%), pneumonia(6%), nausea(4%), AST increased(3%), hypophosphataemia(3%)	(61)
Camrelizumab(n=228)	ESCORT	III	Esophageal squamous cell carcinoma	**Total:94%** RECCP(80%), hypothyroidism(17%), anaemia(11%), asthenia(10%), WBC decreased(7%), diarrhea(6%), decreased appetite(5%), NEU decreased (4%)	**Total:19%** Anaemia(3%), diarrhea(1%), lymphocyte count decreased(1%), hyponatraemia(1%), death(1%)	(62)
Tislelizumab plus platinum and pemetrexed(n= 223)	RATIONALE 304	III	Nonsquamous NSCLC	Anemia(86%), lukopenia(82%), neutropenia(82%), thrombocytopenia(70%), ALT increased (48%), nausea(43%), AST increased(43%), fatigue(38%), decreased appetite(34%), vomiting(27%), musculoskeletal pain(25%)	Neutropenia(44%), leukopenia(22%), thrombocytopenia(19%), anemia(15%), increased ALT(4%), increased AST(2%), fatigue(1%), decreased appetite(1%)	(63)
Tislelizumab(n=256)	RATIONALE 302	III	Esophageal Squamous CellCarcinoma	AST increased(11%), anemia(11%), hypothyroidism(10%), fatigue(7%), decreased appetite(6%), diarrhea(5%), asthenia(5%), malaise(4%), weight decreased(3%), nausea(3%), leukopenia(3%)	/	(64)
Tislelizumab plus paclitaxel and carboplatin(n=120)	RATIONALE 307	III	Squamous NSCLC	Anemia(88%), alopecia(64%), NEU decreased(63%), WBC decreased(53%), leukopenia(48%), decreased appetite(43%), neutropenia(43%), ALT increased(42%), AST increased(36%), PLT decreased (34%)	NEU decreased(52%), neutropenia(33%), WBC decreased(23%), leukopenia(16%), anemia(8%), thrombocytopenia(6%), rash(3%), pain in extremity(3%), ALT increased(2%)	(65)
Tislelizumab plus nab-paclitaxel and carboplatin(n=118)	RATIONALE 307	III	Squamous NSCLC	Anemia(93%), alopecia(69%), NEU decreased(61%), WBC decreased(58%), leukopenia(56%), decreased appetite(44%), PLT decreased (44%), neutropenia(42%), ALT increased(35%), AST increased(34%)	NEU decreased(46%), WBC decreased(27%), neutropenia(27%), leukopenia(25%), anemia(23%), PLT decreased (14%), thrombocytopenia(13%), AST increased(2%), rash(2%), decreased appetite(1%)	(65)
Penpulimab(n=85)	AK105-201	II	Classical Hodgkin lymphoma	Hypothyroidism(35%), upper respiratory tract infection(28%), fever(27%), ALT increased(26%), hypertriglyceridemia(21%), reduced leucocyte count(20%), rash(18%), AST increased(16%), anemia(16%), elevated TSH(15%)	Skin rash(4%), hyperlipidemia(4%), NEU decreased(2%), weight gain(1%), fever(1%), hypertrigly(1%), reduced leucocyte count(1%), anemia(1%)	(66)
Penpulimab plus carboplatin and paclitaxel(n=175)	AK105-302	III	Squamous NSCLC	/	**Total:63.6%**	(67)
Zimberelimab(n=85)	GLS-010-cHL	II	Classical Hodgkin lymphoma	Pyrexia(32%), hypothyroidism(21%), NEU decreased(20%), ALT increased(20%), WBC decreased(19%), weight increased(13%), blood bilirubin increased(12%), upper respiratory tract infection(11%), pruritus(11%), anemia(11%), AST increased(11%), hepatic function abnormal(11%)	Hepatic function abnormal(6%), hyperuricemia(5%), weight increased(4%), NEU decreased(4%), upper respiratory tract infection(2%), hypertriglyceridemia(2%), pyrexia(1%), lymphocyte count decreased(1%), hypokalemia(1%)	(68)
Serplulimab plus cisplatin and 5-fuorouracil(n=382)	Serplulimab-ESCC	III	Esophageal squamous cell carcinoma	**Total:99%** Anemia(76%), nausea(64%), WBC decreased(58%), NEU decreased(56%), PLT decreased(43%), vomiting(43%), appetite decreased(42%), asthenia(30%), blood creatinine increased(16%)	**Total:64%** NEU decreased(19%), anemia(18%), WBC decreased(11%), hyponatremia(5%), hypokalemia(4%), nausea(3%), vomiting(3%), appetite decreased(2%), AST increased(2%)	(69)
Serplulimab plus carboplatin and etoposide(n=389)	ASTRUM-005	III	Extensive-stage SCLC	**Total:70%** Anemia(22%), WBC decreased(20%), PLT decreased(15%), hypothyroidism(15%), nausea(13%), ALT increased(12%), hyperthyroidism(11%), AST increased (10%)	**Total:33%** NEU decreased(14%), WBC decreased(8%), PLT decreased(6%), anemia(5%), neutropenia (4%), leukopenia(3%), decreased lymphocyte count(2%), hyperglycemia(2%)	(70)
Adebrelimab(n=230)	CAPSTONE-1	III	Extensive-stage SCLC	**Total:100%** NEU decreased(95%), WBC decreased(94%), anaemia(85%), PLT decreased(83%), alopecia(44%), ALT increased(41%), nausea(40%), AST increased(35%), decreased appetite(30%), vomiting(26%)	**Total:86%** NEU decreased(76%), WBC decreased(46%), PLT decreased, anaemia(28%), ALT increased(2%), γ-glutamyltransferase increased(2%), decreased appetite(2%), hyponatraemia(2%), hypokalaemia(2%)	(71)
Atezolizumab(n=286)	IMpower110	III	NSCLC	**Total:90%** Anemia(15%), decreased appetite(15%), nausea(14%), asthenia(13%), fatigue(13%), constipation(12%), hyponatremia(6%), pneumonia(5%), hyperkalemia(4%)	**Total:34%** Anemia(2%), hyponatremia(2%), pneumonia(2%), hyperkalemia(2%), decreased appetite(1%), asthenia(1%), fatigue(1%), constipation(1%), neutropenia(1%)	(72)
Atezolizumab plus nab-paclitaxel(n=453)	IMpassion130	III	Triple-negative breast cancer	**Total:93%** Alopecia(57%), fatigue(47%), nausea(46%), diarrhea(32%), anaemia(28%), constipation(26%), cough(25%), headache(24%), neuropathy peripheral(22%), neutropenia(21%)	**Total:50%** Neutropenia(8%), neuropathy peripheral(6%), NEU decreased(5%), fatigue(4%), anaemia(3%), peripheral sensory neuropathy(2%), AST increased(2%), hypokalaemia(2%), pneumonia(2%), diarrhea(2%)	(73)
Atezolizumab plus carboplatin and etoposide(n=198)	IMpower133	III	Extensive-stage SCLC	**Total:95%** Rash(20%), hypothyroidism(13%), hepatitis(8%), Infusion-related reactions(6%), hyperthyroidism(6%), pneumonitis(3%), colitis(2%)	**Total:59%**	(74)
Atezolizumab plus bevacizumab(n=329)	IMbrave150	III	Hepatocellular carcinoma	Proteinuria(29%), hypertension(28%), AST increase(16%), fatigue(16%), pruritus(14%), ALT increase(12%), decreased appetite(12%), diarrhea(11%), infusion-related reaction(11%), PLT decreased(10%), hypothyroidism(10%), rash(10%)	Hypertension(12%), AST increase(5%), proteinuria(4%), PLT decreased(2%), fatigue(2%), ALT increase(2%), infusion-related reaction(2%), pneumonia(1%), gastrointestinal hemorrhage(1%), liver injury(1%), decreased appetite(1%), diarrhea(1%)	(75)
Durvalumab plus tremelimumab and platinum–etoposide(n=266)	CASPIAN	III	Extensive-stage SCLC	**Total:89%** Neutropenia(43%), anaemia(38%), nausea(32%), alopecia(30%), decreased appetite(21%), constipation(20%), thrombocytopenia(20%), fatigue(20%), asthenia(14%), vomiting(14%)	**Total:64%** Neutropenia(32%), anaemia(13%), thrombocytopenia(9%), leucopenia(6%), febrile neutropenia(6%), hyponatraemia(5%), pneumonia(5%), diarrhea(3%)	(76)
Durvalumab plus platinum–etoposide(n=265)	CASPIAN	III	Extensive-stage SCLC	**Total:98%** Neutropenia(42%), anaemia(38%), nausea(34%), alopecia(32%), decreased appetite(18%), fatigue(18%), constipation(17%), asthenia(16%), thrombocytopenia(15%), vomiting(15%), leucopenia(15%)	**Total:65%** Neutropenia(24%), anaemia(9%), thrombocytopenia(6%), leucopenia(6%), NEU decreased(6%), febrile neutropenia(5%), hyponatraemia(4%), hypertension(3%), lipase increased(3%)	(76)
Durvalumab(n=475)	PACIFIC	III	Stage III SCLC	**Total:97%** Cough(35%), pneumonitis or radiation pneumonitis(34%), fatigue(24%), dyspnea(22%), diarrhea(18%), pyrexia(15%), decreased appetite(14%), nausea(14%), pneumonia(13%), arthralgia(12%)	**Total:30%** Pneumonia(4%), pneumonitis or radiation pneumonitis(3%), anemia(3%), dyspnea(1%), diarrhea(1%), asthenia(1%), musculoskeletal pain(1%)	(77)
Durvalumab plus tremelimuma (n=388)	HIMALAYA	III	Hepatocellular Carcinoma	Rash(32%), diarrhea(27%), fatigue(26%), pruritus(23%), musculoskeletal pain(22%), abdominal pain(20%), decreased appetite(17%), hypothyroidism(14%), pyrexia(13%), nausea(12%), insomnia (10%)	Diarrhea(6%), fatigue(3.9%), rash(2.8%), musculoskeletal pain(2.6%), abdominal pain(1.8%)	(78)
Durvalumab plus tremelimuma+chemotherapy(n=338)	POSEIDON	III	NSCLC	**Total:92.7%** Anemia(43.6%), nausea(37.6%), neutropenia(29.1%), decreased appetite(20.9%), fatigue(19.7%), thrombocytopenia(19.7%), rash(15.8%), vomiting(14.2%), diarrhea(13.9%), leukopenia(12.7%)	**Total:51.8%** Anemia(17.3%), neutropenia(16.1%), neutrophil count decreased(7.3%), thrombocytopenia(5.5%), leukopenia(2.7%)	(79)
Avelumab(n=344)	JAVELIN Bladder 100	III	Urothelial carcinoma	**Total:98%** Fatigue(18%), pruritus(17%), urinary tract infection(17%), diarrhea(17%), arthralgia(16%), asthenia(16%), constipation(16%), back pain(16%), nausea(16%), pyrexia(15%)	**Total:47%** Urinary tract infection(4%), anemia(4%), fatigue(2%), hematuria(2%), diarrhea(1%), arthralgia(1%), constipation(1%), back pain(1%), vomiting(1%), infusion-related reaction(1%)	(80)
Avelumab plus axitinib(n=434)	JAVELIN Renal 101	III	Renal cell carcinoma	**Total:100%** Diarrhea(62%), hypertension(50%), fatigue(41%), nausea(34%), PPES (33%), dysphonia(31%), decreased appetite(26%), hypothyroidism(25%)	**Total:71%** Hypertension(26%), diarrhea(7%), fatigue(3%), PPES (6%),ALT increased (6%), AST increased(4%), fatigue(3%)	(81)
Envafolimab(n=103)	Envafolimab	II	dMMR/MSI-H solid tumors	WBC decreased(17%), asthenia(17%), rash(16%), hypothyroidism(16%), hyperthyroidism(12%), NEU decreased(12%), anemia(12%)	Anemia(5%), NEU decreased(1%), rash(1%),	(82)
Sugemalimab plus carboplatin and paclitaxel(n=320)	GEMSTONE-302	III	NSCLC	**Total:100%** Anaemia(73%), NEU decreased(58%), WBC decreased(56%), PLT decreased(33%), AST increased(33%), ALT increased(32%), appetite decreased(23%), nausea(22%), alopecia(19%), asthenia(16%)	**Total:64%** NEU decreased(33%), WBC decreased(14%), anaemia(13%), PLT decreased(10%), neutropenia(4%), γ-glutamyltransferase increased(2%), leukopenia(2%), hepatic function atypical(2%), pneumonia(2%)	(83)
Sugemalimab(n=255)	GEMSTONE-301	III	NSCLC	**Total:76%** Pneumonitis(19%), hypothyroidism(17%), hyperthyroidism(15%), ALT increased(13%), AST increased(12%), rash(7%), pruritus(6%), anaemia(5%), GGT increased(5%), hypertriglyceridaemia(4%), blood cholesterol increased(4%)	**Total:10%** Pneumonitis(3%), pneumonia(2%), hypothyroidism(1%), rash(1%), hypertriglyceridaemia(1%)	(84)
Cadonilimab(n=111)	AK104-201	II	Cervical cancer	**Total:96.4%** Anaemia(7.2%), decreased appetite (2.7%)	**Total:28.8%**	(85)

NSCLC, non-small cell lung cancer; SCLC, small cell lung cancer; dMMR, defective mismatch repair; MSI-H, microsatellite instability high; ALT, alanine aminotransferase; AST, aspartate aminotransferase; WBC, white blood cell count; PLT, platelet count; NEU, neutrophil count; TSH, thyroid stimulating hormone; RECCP, reactive cutaneous capillary endothelial proliferation; PPES, palmar-plantar erythrodysesthesia syndrome; GGT, gamma-glutamyltransferase.

### 3.1 Immune-related dermatologic adverse events

Dermatologic irAEs are commonly observed in patients, impacting up to 50% of individuals. Most cases of dermatologic irAEs are mild reactions. The frequently reported dermatologic irAEs consist of erythema, rash, pruritus, reactive cutaneous capillary endothelial proliferation (RCCEP), and vitiligo (86). Numerous studies on camrelizumab have consistently identified RCCEP as an adverse event, with an incidence rate as high as 80% even when used as monotherapy (59, 61, 62). Nevertheless, severe cases (grade 3-5) of RCCEP are infrequent, occurring in less than 2% of patients. The rash can manifest with various clinical characteristics such as maculopapular or erythematous lesions. Data have indicated that the occurrence of rash in patients receiving nivolumab and pembrolizumab ranges from 34% to 40% (87). However, the risk of rash significantly increases when ipilimumab is combined with these drugs, and the overall prevalence of vitiligo is 8% (88–90). According to findings from CheckMate 914 (43), the incidence rate of rash in the treatment of Renal cell carcinoma with nivolumab plus ipilimumab was reported to be 21%. It is important to note that another treatment regimen containing CTLA-4 inhibitors has a higher rash incidence. In the HIMALAYA study (78), the safety of durvalumab plus tremelimumab in the treatment of hepatocellular carcinoma (HCC) is currently under investigation, and the reported incidence of rash is 32%.

Dermatological irAEs typically arise during the initial two weeks of therapy and can be observed in any patient with cancer. Less frequently occurring dermatologic irAEs entail actinic keratosis and skin exfoliation, along with dermatitis acneiform, dry skin, and palmar-plantar erythrodysesthesia syndrome (PPES) (41, 51). Patients exhibiting grade 1 dermatologic irAEs, as stipulated by the Common Terminology Criteria for Adverse Events 5.0, are eligible for ICI treatment. However, in the event of a grade 3 rash, it becomes imperative to introduce prednisone, a systemic steroid, at a daily dose of 0.5–1 mg/kg and temporarily suspend ICI treatment (91). The primary approach to managing dermatologic irAEs involves providing supportive care. Utilizing medium to high-potency topical corticosteroids proves beneficial for treating the rash. Alternatively, pruritus symptoms can be relieved by using cold compresses, oatmeal baths, and systemic antihistamines such as hydrochloride and hydroxyzine hydrochloride (92). As a rule, RCCEP generally does not necessitate specialized treatment nor is it affected by GSCs. The majority of symptoms tend to spontaneously resolve within approximately 1.6 months after discontinuing camrelizumab. For large nodules and instances of bleeding, it is crucial to implement measures to promote hemostasis and prevent infection (93).

### 3.2 Immune-related endocrinopathies adverse events

Thyroid disorders, hypophysitis, insulin-deficient diabetes mellitus, and primary adrenal insufficiency (PAI) have been cited as irAEs caused by ICIs therapy (94). Most instances of thyroid irAEs present as painless thyroiditis accompanied by temporary thyrotoxicosis (95). In patients with severe thyrotoxicosis, there is often a subsequent period of hypothyroidism. Over 40% of patients experience permanent hypothyroidism and necessitating thyroid hormone replacement (96). Some individuals may develop primary hypothyroidism without prior thyrotoxicosis (95). Two observational studies examining thyroid irAEs found that between 42-53% of patients encountered immune checkpoint inhibitor-related thyroid irAEs (96, 97). The incidence of thyroid dysfunction in patients treated with a combination of PD-L1 inhibitors and CTLA-4 inhibitors has been reported as high as 56% (98). Research suggests that hypophysitis is frequently associated with CTLA-4 inhibitors, whereas PD-1 inhibitors are more commonly linked to thyroid dysfunction in comparison to PD-L1 inhibitors (95, 98). A clinical trial investigating zimberelimab for the treatment of classical Hodgkin lymphoma discovered a 21% incidence rate of hypothyroidism (68). Conversely, a phase 3 clinical study on sugemalimab as monotherapy in NSCLC reported a 17% incidence of hypothyroidism (84). PAI poses a significant clinical concern. The analysis of the 2020 WHO VigiBase report revealed immune-related PAI to be linked to a considerable level of morbidity, with over 90% of cases categorized as severe, the mortality rate was observed to be 7.3% (99).

Most cases of immune-related endocrinopathies typically occur within 12 weeks of initiating ICIs therapy. However, there have been reports of some endocrinopathies developing several months to years after starting ICIs treatment (100). A retrospective study (101) found that 67% of patients did not show any symptoms during the thyrotoxicosis phase, which lasted approximately 6 weeks. After around 10.4 weeks, 84% of patients developed hypothyroidism. The majority of immune-related thyroid complications are mild to moderate, and thyrotoxicosis only requires active surveillance without treatment (102). It is recommended to regularly monitor thyroid function, including levels of thyroid-stimulating hormone and free thyroxine after completing 5-6 cycles of ICIs treatment (103). Symptoms of hyperthyroidism can be alleviated by orally administering receptor blockers such as propranolol, metoprolol, or atenolol (104). When thyroid-stimulating hormone levels exceed 10 mIU/L, treatment with levothyroxine is recommended. Typically, levothyroxine is initiated at a low dose of 25~50 µg/day or 1.6 µg/kg (102, 105). For overt hypothyroidism, levothyroxine is usually initiated at a low dose of 25-50 μg/day (106). However, in young and healthy patients, it may also be initiated at a full estimated replacement dose of 1.6 g/kg body weight (107). In elderly patients or those with heart disease, it is particularly important to initiate treatment with a lower initial dose of 12.5~25 μg/day and titrate slowly (107). In cases of a patient developing an acute adrenal crisis or severe illness, it is crucial to promptly administer stress doses of GCSs. Additionally, mineralocorticoid replacement therapy is necessary for the treatment of PAI. It is important to note that endocrine irAEs are often irreversible and may require lifelong hormone replacement therapy (92).

Regarding to immune-related diabetes, patients commonly display symptoms and indications of hyperglycemia or diabetic ketoacidosis (DKA) (102). Although rare, diabetes mellitus and PAI are endocrine toxicities that can be life-threatening if not promptly recognized and treated. A study conducted by Kotwal A. et al. (108) discovered that just 1.4% of patients who received treatment with ICIs for more than 6 years developed new-onset insulin-dependent diabetes or experienced significant deterioration of type 2 diabetes. Nevertheless, clinical trials have reported a slightly higher incidence rate, with hyperglycemia observed in 6% of patients treated solely with serplulimab (70). Another recent study revealed a noteworthy correlation between the utilization of metformin to regulate blood glucose levels and a 53% heightened risk of mortality following ICIs treatment (109). Hence, vigilant monitoring of blood glucose levels post-ICI usage is imperative to promptly detect ICI-related diabetes and prevent DKA (102). Moreover, it is essential to rule out the presence of ketoacidosis. When blood glucose levels are raised, promptly assessing glycosylated hemoglobin levels, and seeking consultation from an endocrinologist is recommended (34, 110).

### 3.3 Immune-related gastrointestinal adverse events

Gastrointestinal irAEs related to the digestive system, such as gastritis, colitis, and enterocolitis, typically manifest themselves approximately 6 to 8 weeks after starting treatment with ICIs (33). Symptoms affecting the upper digestive tract nausea, vomiting, dysphagia, pain in the upper abdomen. On the other hand, manifestations in the lower digestive tract can involve abdominal pain, hematochezia, constipation, and diarrhea (111). There have been instances where diarrhea and/or colitis may develop months after discontinuing immunotherapy, resembling symptoms similar to chronic inflammatory bowel disease (34). Among the various gastrointestinal irAEs associated with immune checkpoint inhibitors, colitis is the most common occurrence during CTLA-4 inhibitor therapy (112). Colitis tends to appear earlier, exhibit greater severity, and frequently necessitates discontinuation of medication. The reported incidence rates of colitis with CTLA-4 inhibitors and PD-1 inhibitors are approximately 27-54% and 19.2%, respectively (113). When both therapies are administered in combination, the incidence rate increases to 44.1% (88).

A study evaluating the safety of toripalimab in combination with gemcitabine and cisplatin (GP) treatment for advanced nasopharyngeal carcinoma reported incidence rates of nausea (69%), vomiting (67%), decreased appetite (53%), and constipation (39%) (53). In a clinical trial that examined the safety of combining avelumab and axitinib for advanced renal cell carcinoma, diarrhea emerged as a frequent side effect, with a reported incidence rate of 62% (81). Similarly, the KEYNOTE-048 study observed a high prevalence of gastrointestinal disorders (83%) in the pembrolizumab and chemotherapy group, wherein constipation was reported in 37% of cases. In comparison, the incidence of gastrointestinal disorders was lower at 57% in the pembrolizumab monotherapy, with constipation also reduced to 20% (50).

The incidence rates of colitis with CTLA-4 inhibitors and PD-1 inhibitors are 27-54% and 19.2%, respectively (113). When these therapies are combined, the incidence rate rises to 44.1% (88). A meta-analysis conducted by Wang DY. et al (114) investigated the incidence of immune-related colitis in patients with solid tumors. The study discovered that ICIs monotherapy with exhibited a 1.3% lower incidence of colitis (any grade) compared to alternative treatments. Severe colitis and severe diarrhea rates were 0.9% and 1.2%, respectively. However, the combination therapy of ipilimumab and nivolumab showed an increase in immune-related colitis (13.6%), severe colitis (9.4%), and severe diarrhea (9.2%). Another meta-analysis conducted in China (115), including more recent clinical trials, concluded that ICIs inhibitors posed a heightened risk of colitis across all grades when compared to chemotherapy. Notably, a solitary patient experienced bloody diarrhea after taking the 70th dose of nivolumab, suggesting a potential association between long-term nivolumab use and immune-related colitis (116). Moreover, reports suggest that raising the dosage of nivolumab or adding osimertinib after long-term stabilization of NSCLC can induce immune-related colitis (117–119).

Patients with grade 1 symptoms can be treated conservatively with a bland diet and oral hydration during episodes of acute diarrhea. For patients presenting with grade 2 symptoms, characterized by moderate diarrhea, it is recommended to start with immunotherapy cessation and initiate corticosteroid treatment as the primary approach. The dosing regimen involves administering oral prednisone or methylprednisolone at a dose of 1 mg/kg/day. If there is no improvement within 2-3 days, the corticosteroid dose should be increased to 2 mg/kg/day. In patients with more severe symptoms (grade 3 and above), the first step is to discontinue immunotherapy and then initiate intravenous methylprednisolone at a dose of 2 mg/kg/day. In cases where there is a persistent lack of response, the addition of a single dose of infliximab should be considered and starting with an initial dose of 5 mg/kg/day (34). Generally, most gastrointestinal irAEs can be effectively managed, but colitis often leads to discontinuation of therapy. When considering the reintroduction of immunotherapy after gastrointestinal irAEs, it is crucial to evaluate the risks on an individual basis (35). Once there is an improvement in grade 2/3 diarrhea, immunotherapy can be resumed. However, if the irAEs are graded as G4, it is advisable to permanently discontinue the therapy (120).

### 3.4 Immune-related hepatic adverse events

Hepatic irAEs can occur at any time after the initial administration of ICIs, but they are most commonly observed between 8 to 12 weeks of starting the therapy. The main indicators of hepatic irAEs are increased levels of alanine aminotransferase (ALT) and/or aspartate aminotransferase (AST), with or without elevation in bilirubin. Patients may experience non-specific symptoms such as fever, fatigue, anorexia, and nausea. Elevation in bilirubin levels can lead to jaundice in the skin and sclera, as well as the presence of tea-colored urine (121). The occurrence of hepatic irAEs is more frequent in patients receiving combination therapy than in those undergoing monotherapy. The incidence of hepatic irAEs varies significantly depending on the type of ICIs, combination therapy, and tumor type (122).

Statistics have indicated that CTLA-4 inhibitors had a higher risk of hepatotoxicity, whereas PD-1 inhibitors appear to be associated with a lower risk (123). Patients with HCC who underwent ICIs therapy also had a higher incidence of ALT/AST elevation compared to patients with another solid tumor (124). Notably, when bevacizumab was combined with sintilimab and atezolizumab in the treatment of HCC, the incidence of AST elevation was 16% and 36%, respectively (58, 75). The ORIENT-32 study also reported a 29% increase in bilirubin levels in the blood. In a meta-analysis of non-HCC patients in the Chinese population (125), who underwent treatment with pembrolizumab, nivolumab, camrelizumab, toripalimab, tislelizumab, and sintilimab, the incidence of any grade of hepatic irAEs ranged from 7.4% to 14.0%. Monotherapy demonstrated an incidence rate of 6.9% to 13.1%, while combination therapy ranged from 12.2% to 37.8% (125).

The standard management of grade 1~2 hepatic dysfunction generally involves close monitoring to detect any worsening liver tests that may indicate a grade 3~4 irAEs at an early stage (126). In cases of grade 3~4 liver toxicity, high-dose intravenous glucocorticoids are administered for 24~48 hours, followed by an oral steroid taper with prednisolone at a dosage of 1~2 mg/kg over a minimum period of 30 days (127). It is recommended to wait until the liver function tests return to at least grade 1 before resuming immunotherapy. Unlike autoimmune hepatitis, hepatic irAEs occur when initiating higher doses of GSCs for a shorter duration, which does not require additional immunosuppression and retreatment with ICIs is not associated with relapse (128). If liver function tests do not improve or worsen within 48 hours of systemic steroid use, alternative medications such as mycophenolate mofetil (500 mg every 12 hours) or infliximab (5mg/kg/day) may be considered (129, 130). A case study reports some success with the use of mycophenolate mofetil in GSCs-refractory cases (131). Give additional doses of infliximab only if there is no improvement after the initial dose (132). However, caution should be exercised when using infliximab as it may increase the risk of severe liver injury (133).

### 3.5 Immune-related pulmonary adverse events

Pulmonary irAEs often manifest with symptoms such as dyspnea, cough, fever, or chest pain. While hypoxia is rare, approximately one-third of patients remain asymptomatic and only show abnormalities on imaging (134, 135). These events typically occur around 2.8 months after starting treatment, and most patients experience grade 1 to 2 symptoms (35). In a phase 3 trial of durvalumab in patients with stage III NSCLC, a high incidence of pneumonitis or radiation pneumonitis (including acute interstitial pneumonitis, interstitial lung disease, and pulmonary fibrosis) was reported, with pneumonia accounting for 13.1% of cases (77). A retrospective study of 205 NSCLC patients found that the incidence of immune-related pneumonia was 19% (136). It has been observed that patients with chronic immune-related pneumonia consistently show lymphocytosis in bronchoalveolar lavage fluid from the initial onset and throughout the steroid taper. Immunofluorescence has revealed rapid infiltration of CD8+ cells (137). Furthermore, patients with pre-existing pulmonary fibrosis have a higher risk of developing anti-PD-1-associated pneumonia (138). Additionally, an increase in blood absolute eosinophil count has been linked to a higher risk of immune-related pneumonitis (139).

Treatment of immune-related pneumonia includes discontinuing ICIs, systemic steroids, and immunosuppressive medications (140). Research indicates that 20% of cases experience a recurrence of immune-related pneumonia upon resuming ICIs (141). Moreover, some patients have developed recurrent pneumonia even after cessation of systemic steroid therapy and without resuming ICIs treatment (142). GSCs remain the primary treatment, and it is crucial to continue preventive measures against the recurrence of pulmonary irAEs for at least 4 weeks, followed by a gradual reduction. It is also important to consider measures to prevent fungal infection and osteoporosis. If a course of corticosteroid therapy fails to alleviate the severity of initial symptoms, the option of immunosuppression with infliximab may be considered (143).

### 3.6 Immune-related hematologic adverse events

Hematologic irAEs include hemolytic anemia, immune thrombocytopenia, lymphopenia, neutropenia, and aplastic anemia (144). These events typically occur around 10 weeks after starting ICIs therapy and can manifest at any time during treatment (145). Data from VigiBase revealed that immune thrombocytopenia had a median onset time of 41 days, while autoimmune hemolytic anemia had a median onset time of 55 days (146, 147). In a retrospective analysis by Kramer R. et al (148), involving 7,626 patients from 18 international cancer centers, hematologic irAEs were reported in 50 patients (0.6%). A meta-analysis of 47 separate studies with 9,324 patients reported that the incidence of anemia was 9.8% in grade, with grades 3 to 5 observed in 5% of cases (149). Although the reported rates of hemolytic anemia, aplastic anemia, and thrombocytopenia are relatively low, it is important to recognize that these conditions can lead to life-threatening situations, as evidenced by documented fatal cases (150–152). In the CAPSTONE-1 study conducted on patients with advanced small cell lung cancer receiving adebrelimab, a notably high incidence of hematological irAEs was observed. Approximately 95% of the patients experienced neutropenia, 94% experienced leukopenia, 85% experienced anemia, and 82% experienced thrombocytopenia (71).

Effective management is crucial in dealing with hematological irAEs. The diagnosis of immune thrombocytopenia can be challenging, and clinicians must be vigilant for symptoms such as easy bruising, petechiae, and spontaneous mucocutaneous bleeding. It is essential for patients to promptly report any of these symptoms (153). While steroids are commonly used to treat mild thrombocytopenia, they may not be sufficient for severe cases (152). Other available treatment options include recombinant human thrombopoietin (TPO), platelet transfusions for short-term and concurrent therapy, intravenous immunoglobulin (IVIG), and the utilization of immunosuppressants like azathioprine and rituximab.

In cases of steroid resistance, TPO receptor agonists such as eltrombopag, herombopag, or avatrombopag can be administered (154). An in-depth and descriptive observational study (144) revealed that 78% of immune-related thrombocytopenia cases were classified as grade 4. All patients underwent steroid treatment, with 67% of them also receiving IVIG. However, 22% of patients did not respond to these treatments and required replacement therapy involving a TPO receptor agonist or rituximab. The study also provided preliminary safety data on rechallenging patients with ICIs. Among the patients, 67% discontinued halted the use of ICIs treatment, while 33% were rechallenged. Out of this group, 33% experienced a relapse of immune-related thrombocytopenia. Currently, the optimal treatment for hematologic irAEs is still under investigation.

### 3.7 Immune-related cardiovascular adverse events

Cardiovascular irAEs can manifest in various ways, including myocarditis, pericarditis, arrhythmias, reduced ventricular function, vasculitis, venous thromboembolism, cardiac valvulitis, and pulmonary hypertension. Myocarditis is characterized by symptoms such as palpitations, chest pain, acute or chronic heart failure, pericarditis, and pericardial effusion (155).

A retrospective study (156) conducted in the United States involved 105 patients from 8 medical centers. The study revealed that the median onset time of immune-related myocarditis after immunotherapy was 27 days. The age of symptom onset was 65 years, and the estimated occurrence rate was 1.9%. Approximately 81% of cases occurred within the first three months of ICIs therapy. Similar results were found in a retrospective analysis conducted in China (157), which involved 2373 individuals receiving ICI monotherapy or combination therapy from 12 medical centers. The estimated event rate of immune-related myocarditis was 1.05%, but the median time of development was delayed to 38 days. Another real-world investigation (158), that included 2647 patients treated with ICIs, revealed cardiovascular irAEs in 89 patients (3.4%), with myocarditis accounting for approximately. 37.1% of cases. Despite immune-related myocarditis being generally rare, it is considered one of the most perilous irAEs due to its high fatality rate, ranging from 27% to 60% (134, 159). For instance, a study on ipilimumab–nivolumab combination therapy reported a mortality rate of 60% in cases of myocarditis (160).

The likelihood of cardiovascular events has been found to triple in cancer patients due to atherosclerosis (161). Furthermore, the combination of PD-1/PD-L1 inhibitors with CTLA-4 inhibitors is also associated with higher rates of cardiovascular irAEs. These irAEs exacerbate the condition, leading to earlier symptom manifestation and increased risk of mortality (162). The increase in cardiac biomarkers is strongly correlated with disease severity and frequently occurs before the onset of symptoms (163). Diagnostic tests primarily involve troponin measurement and electrocardiogram, while cardiac magnetic resonance imaging and endomyocardial biopsy are deemed the gold standard for diagnosis (164). Treatment options are determined based on risk stratification.

Palaskas NL. et al. (165) demonstrated that some patients with low-grade myocardial inflammation may continue ICIs treatment without immunosuppressive therapy. The first-line treatment suggests different doses of GSCs, while the second-line treatment includes the use of immunosuppressants such as IVIG and anti-thymocyte globulin. It should be noted that the second-line treatment is recommended for life-threatening situations or when GSCs are ineffective (166). However, high-dose infliximab should be avoided in patients with moderate to severe heart failure. Unlike other irAEs, restarting ICIs has been reported to be extremely dangerous (167).

### 3.8 Immune-related neurologic adverse events

Neurological irAEs demonstrate significant heterogeneity and occur relatively infrequently. These events can affect both the central and peripheral nervous systems, leading to conditions such as myositis, neuropathy, encephalopathy, and myasthenia gravis (38). Several phase 3 clinical trials have identified a higher occurrence of neurological irAEs. For instance, in patients with advanced nasopharyngeal carcinoma treated with toripalimab combined with GP, the incidence of peripheral neuropathy was 30%. Similarly, in patients treated with toripalimab, paclitaxel, and cisplatin for advanced esophageal squamous cell carcinoma, the incidence of peripheral neuropathy was 40% (53, 54). In a clinical trial (65) investigating the combination of tislelizumab, paclitaxel and carboplatin for advanced NSCLC, the occurrence rate of hypoesthesia was reported to be 23%, notwithstanding the inclusion of both immunotherapy and chemotherapy in these treatment regimens. A comprehensive meta-study (168) merging data from 59 clinical trials revealed that neurological irAEs were documented in 6% of patients receiving PD-1 inhibitors, with the majority categorized as grade 1-2. Headache was the most frequently reported symptom, while grade 3 or higher neurological irAEs were observed in less than 1% of cases. Additional studies (169, 170) have reported estimated incidences of neurological irAEs ranging from approximately 1% to 12% in patients undergoing immunotherapy, primarily occurring within the initial 6 months of commencing ICIs. Furthermore, the peripheral nervous system is found to be more susceptible to these adverse events compared to the central nervous system.

To establish a conclusive link between peripheral neuropathy and ICIs, it is crucial to assess alternative potential origins in patients suspected of having neuropathy. It should be noted that these symptoms might also arise from other medications (171). Several factors should be considered when ruling out other possible causes, including the duration of drug use, presence of pre-existing neurological conditions, simultaneous irAEs and overlapping syndromes, and improvement upon discontinuation of the drug and/or initiation of GSCs (172). In addition, alternative immunomodulatory approaches, such as antirheumatic drugs, should be taken into account as well (173).

### 3.9 Immune-related musculoskeletal adverse events

Patients treated with ICIs have reported experiencing arthralgia and myalgia; however, there has not been a comprehensive report on the incidence of mild to moderate arthritis (174). According to a study, 13.3% of patients receiving PD-1 inhibitors experienced arthralgia, with a median onset time of 100 days. Specifically, arthralgia was observed in 18% of patients with advanced cutaneous squamous cell carcinoma who received cemiplimab monotherapy (51). In a study by Cappelli LC. et al (175) data from 52 trials of musculoskeletal irAEs revealed that arthritis was reported arthritis in 1–43% and myalgia in 2–20% of patients across 5 out of 33 clinical trials. To manage symptoms of myalgia or joint pain, nonsteroidal anti-inflammatory drugs (NSAIDs) or corticosteroids are generally recommended. Once symptoms improve to grade 1 or less, it is wise to gradually reduce the dose of corticosteroids over 4-6 weeks. If the corticosteroid dose cannot be reduced to 10 mg per day within 6-8 weeks, further consideration of antirheumatic drugs is recommended. Patients who experience symptoms persisting for more than 6 weeks or need a daily corticosteroid dose exceeding 20 mg that cannot be reduced to less than 10 mg daily within 4 weeks, should consult with a rheumatologist (176). In most patients, symptoms improved with the use of NSAIDs, while low-dose GSCs were required by 23.1% of patients and 7.6% required additional immunosuppressive therapy (177).

### 3.10 Other immune-related adverse events

In this section, other irAEs will also be discussed, including immune-related infusion reactions, ocular adverse reactions, and nephrotoxicity.

Infusion reactions related to ICIs are typically characterized by symptoms such as low-grade fever, chills, headache, or nausea, which can be ascribed to the nonspecific release of cytokines (178). A study involving patients with advanced renal cell carcinoma who received the combination of avelumab and axitinib, reported infusion reactions in 12.2% of patients, with grade 3 or higher reactions observed in 1.6% of cases (81). Manifestations of infusion reactions are usually mild, and mild fever and chills can be managed with NSAIDs. In certain cases, it may be advisable to consider reducing the dosage or discontinuing the infusion (34, 179).

The incidence of ocular irAEs is exceedingly low, less than 1%, and typically manifests within six months of ICI utilization (180). Ophthalmoplegia and uveitis are more prone to appear within the initial 10 weeks, while dry eye and other ocular irAEs may develop later (181). Among lung cancer patients receiving ICIs, the most prevalent ocular irAEs were ophthalmoplegia (40.51%), uveitis (20.25%), and dry eye syndrome (17.72%). Uveitis can usually be effectively treated with topical corticosteroids applied to the surface of the eye, although severe cases may necessitate GSCs administered throughout the body. Other treatment options include using subconjunctival GSCs, injecting dexamethasone directly into the eye, and injecting triamcinolone acetonide around the area near the eye (182). Prompt examination is crucial when symptoms of worsening vision, spots in vision, or redness of the conjunctiva appear (183). The occurrence of uveitis does not necessarily require suspension of immunotherapy. Symptomatic treatment of most ocular irAEs demonstrates exceptional and swift responses, with an overall remission rate as high as 92.31% (except for ophthalmoplegia) (184).

Acute kidney injury (AKI) is the common presentation for most cases of immune-related nephrotoxicity. It requires dialysis and results in abnormal levels of electrolytes (185). The median time to onset of immune-related nephrotoxicity usually occurs within a span of 3 to 4 months (186). Among patients receiving PD-1 inhibitors, the combined estimated rate of AKI was 2.2%. Additionally, interstitial nephritis had a combined estimated rate of 16.6% within this group (187). Nevertheless, the reported incidence of AKI may be higher than what is currently known. Evidence from case reports and cohort studies suggests a possibility of 10% to 30% in clinical practice. For instance, a cohort study reported an incidence of 16.5% (188), while real-world population data reported an incidence of 17% (189). It is important to note that patients with immune-related AKI often experience extrarenal toxicities, including rash, thyroiditis, and colitis, ranging from 40%–87% (188, 190, 191). After diagnosing immune-related AKI, clinicians should thoroughly assess the patient’s medication history and discontinue nephrotoxic drugs. Symptomatic treatment usually involves corticosteroids, and if dialysis is required due to renal impairment, ICIs should be immediately discontinued (160).

## 4 Discussion

### 4.1 Association between irAEs and response to treatment

In 2018, a study conducted by Shafqat H. et al. (192) unveiled a connection between the occurrence of irAEs enhanced progression-free survival (PFS) in patients with various tumor types (192). Further investigations have provided additional evidence supporting the potential correlation between irAEs and clinical benefits. For instance, patients who experienced immune-related arthralgia exhibited better treatment responses, characterized by improved PFS and overall survival (OS) (177). Two studies (193, 194) involving lung cancer patients showcased improved clinical outcomes among individuals who encountered irAEs while undergoing nivolumab treatment. These patients exhibited a higher objective response rate (ORR) and increased PFS compared to those without irAEs. Additionally, a multicenter cohort study unveiled a connection between the progression of multisystem irAEs and improved OS (195). Interestingly, patients who developed late irAEs demonstrated a higher ORR than those with early irAEs (196).

A meta-analysis (197) encompassing 4971 subjects from 30 studies discovered a significant correlation between the development of irAEs and improved survival in tumor patients treated with PD-1 inhibitors. Notably, the group of patients who received ICIs as monotherapy showed a more prominent correlation in cancer outcomes compared to the group receiving combination therapy. Another meta-analysis (198) consolidated these findings, affirming a positive association between the occurrence of irAEs and enhancements in ORR, PFS, and OS, regardless of tumor site, type of ICIs, or irAEs status. It should be pointed out that grade 3 or 4 irAEs were associated with improved ORR, yet worse OS. However, a retrospective study reported contradictory findings, claiming that patients with immune-related constipation faced a significantly higher risk of disease progression, but no significant association with OS was observed (199).

### 4.2 Differences in adverse events between PD-1 inhibitors and PD-L1 inhibitors

Initially, Spagnuolo A. et al (200) discovered no significant distinction in irAEs between the two ICIs. Previous research indicates that patients who received PD-1 inhibitors had a higher occurrence of grade 3 or higher irAEs (201) and were more susceptible to pneumonia and thyroiditis (202). Conversely, PD-L1 inhibitors were associated with lower rates of cardiac complications and overall mortality compared to PD-1 inhibitors. They also exhibit a minimal risk of rash, elevated ALT, colitis, and hypothyroidism (203). Out of the 49 clinical trials analyzed (**Table 1**), it can be observed that immunotherapy generally leads to a higher incidence of anemia, neutropenia, leukopenia, and nausea. This pattern is particularly evident in regimens incorporating PD-1 inhibitors. On the other hand, regimens containing PD-L1 inhibitors tend to cause fatigue more frequently. Even when ICIs are administered as monotherapy, it is still observed that PD-1 inhibitor regimens have a higher incidence of anemia, followed by hyperthyroidism. Similarly, patients treated with PD-L1 inhibitors are more prone to experiencing fatigue, pneumonia, and cough. Combination regimens of PD-1/PD-L1 and CTLA-4 inhibitors were associated with higher rates of fatigue, nausea, rash, and diarrhea/colitis. A meta-analysis of clinical studies investigating regimens containing ipilimumab and tremelimumab found that irAEs primarily manifested as skin lesions (rash, pruritus, and vitiligo) and colitis, which aligns with our observed outcomes (204).

In terms of monotherapy, atezolizumab demonstrated a lower overall risk of any grade irAEs compared to pembrolizumab, while avelumab exhibited a lower risk of grade ≥3 irAEs (205). A comprehensive study involving 36 head-to-head phase 2/3 clinical trials revealed differences in the toxicity profiles of different PD-1/PD-L1 inhibitors (206). Specifically, nivolumab was more frequently correlated with endocrine toxicity, pembrolizumab displayed a higher prevalence of arthralgia, pneumonia, and hepatotoxicity, and atezolizumab showed a strong inclination towards symptoms such as hypothyroidism, nausea, and vomiting (206). These studies including CameL, CameL-sq, and ESCORT have confirmed that camrelizumab has a higher tendency to induce RCCEP (59, 61, 62), whereas adebrelimab was reported to give rise to various types of hematological irAEs in CAPSTONE-1 (71). These observations suggest that the pattern of irAEs varies among different PD-1/PD-L1 inhibitors, potentially owing to disparities in their capacity to stimulate immune cells (207). One specific difference to note is that PD-L1 inhibitors do not inhibit the interaction between PD-1 and PD-L2, which plays a role in suppressing the immune response. What’s more, PD-L2 binds to the molecule b, regulating respiratory immunity (208). These factors might account for the discrepancy in the occurrence of particular irAEs between PD-1 inhibitors and PD-L1 inhibitors (209).

### 4.3 Strategies to limit irAEs

With the widespread use of ICIs, oncologists’ understanding and management of irAEs are gradually improving. This review will highlight several strategies to alleviate irAEs.

The first step towards effectively limiting irAEs is to properly profile patients before treatment begins. Additionally, physicians and nurses must have accurate information about patients should serve as early indicators of irAEs. One important strategy is regular monitoring of patients throughout their treatment and during the follow-up period. Close monitoring of control indicators and organ functions is essential for the prompt detection, reporting, and treatment of irAEs (35). For instance, severe cutaneous irAEs, such as pruritus or rash, can signal the presence of other irAEs. Patients with dermatologic irAEs are more susceptible to the occurrence of gastrocolitis, while those with immune-related psoriasis are more prone to endocrine irAEs (210). Furthermore, certain irAEs such as diarrhea and colitis may manifest several months after the cessation of ICIs treatment (211). Therefore, long-term follow-up is crucial, as there is a possibility of delayed onset of pneumonia or skin irAEs (212). Currently, it is recommended to follow up with patients for at least two years after completing ICIs treatment (33).

Secondly, symptomatic treatment plays a crucial role in managing irAEs. GSCs are commonly chosen to treat the main irAEs (35). Based on experience with nivolumab for irAEs, high-dose GSCs should be used cautiously due to potential exceptional reactions, although there are case reports of overall improvement in the condition (213, 214). For grade 1-2 irAEs, oral corticosteroids are typically prescribed. In cases where irAEs affect specific organs such as the heart, lungs, liver, and nervous system, high-dose intravenous GSCs are among the preferred prescriptions for prompt intervention. If GSCs prove to be ineffective, other immunosuppressants such as infliximab, mycophenolate mofetil, tacrolimus, and anti-thymocyte globulin may be taken into account (1). It has been found that glucocorticoid therapy was not necessary for hypothyroidism and other endocrine irAEs (such as diabetes mellitus); replacement hormone therapy is recommended (28, 215).

Thirdly, physicians must consider the possibility of continuation or cessation and subsequent reexposure of ICIs. If patients only experience mild cutaneous or endocrine irAEs, it is acceptable to continue ICIs (87). However, once severe or life-threatening irAEs occur, especially grade 3-4 cardiac, pulmonary, and neurotoxicity, it is imperative to permanently stop the administration of such ICIs (33). If irAEs are downgraded from grade 2 to grade 1, restarting ICIs becomes a viable option (216). Alternatively, replacing ICIs upon reboot is another strategy. An illuminating case report (217) demonstrated that a patient developed immune-related grade 3 colitis, requiring the discontinuation of ipilimumab. However, the patient subsequently received pembrolizumab for over 20 months without experiencing serious irAEs and achieved a partial objective response. When rechallenging with ICIs, it is of utmost importance to closely monitor the reemergence of the initial irAEs (218), as well as the patient’s tumor response status. If irAEs resurface, it is advisable to permanently discontinue the use of such ICIs. A retrospective study (219) discovered that 14% of NSCLC patients had to terminate treatment due to irAEs when using ICIs. Among these patients, 56% were rechallenged with ICIs after the initial treatment. In the re-challenged patient cohort, 48% did not encounter any subsequent irAEs, while 26% experienced a recurrence of the initial irAEs and 26% developed new irAEs.

There is an ongoing debate regarding the best strategies for the management of irAEs. In addition to the previously mentioned mitigation approaches, it is important to consider additional strategies for managing these adverse events. These may include educating patients about their medications, improving guidelines for irAE management, standardizing the reporting of irAEs, and carefully selecting ICIs (220). Furthermore, Sullivan RJ. et al. (7) proposed several key approaches to alleviate irAEs, such as adjusting the dose and administration schedule of ICIs, developing alternative checkpoints, and altering the microbiota. These innovative approaches provide valuable insights for future investigations.

## 5 Conclusion

ICIs can induce unforeseen adverse effects on the body. The emergence and intensity of irAEs are influenced by various immune mechanisms. These mechanisms include the direct destruction of normal cells by monoclonal antibodies, the production of autoantibodies by B cells, T cell activation triggering cytokine pathways, and the influence of gut microbiota.

IrAEs exhibit different clinical manifestations, occurrence times, and impacts on different tissues and organs due to the variations in ICIs and cancer types. Currently, the treatment of irAEs has been mostly empirical, utilizing immune-based approaches for managing primary autoimmune diseases (9). Existing guidelines recommend the use of corticosteroids as the first-line treatment for the most severe forms of irAEs. However, a major limitation of these guidelines is the lack of stratification of irAEs based on the etiology of the immune histopathology (34, 35, 87, 133). While irAEs are generally rare and mostly mild to moderate, there have been cases where serious adverse reactions have resulted in fatal consequences. Therefore, early identification and diagnosis of certain non-specific irAEs, such as cardiac and endocrine irAEs, through regular examinations are crucial. In situations where a wide range of irAEs are present, consultation with experts from various disciplines may be necessary. Nevertheless, further research is required to determine the efficacy of these interventions in reducing the occurrence of irAEs.

## Author contributions

TY: Writing – original draft. LY: Funding acquisition, Writing – review & editing. JZ: Investigation, Supervision, Writing – original draft. YC: Supervision, Visualization, Writing – review & editing. YF: Supervision, Validation, Writing – original draft. JT: Project administration, Resources, Writing – original draft. DL: Writing – original draft, Writing – review & editing.
